# Natural course of diffuse large B cell lymphoma—a manifestation in buccal mucosa

**DOI:** 10.1186/s12957-019-1766-6

**Published:** 2019-12-16

**Authors:** Nishant Batta, Mridula Shukla, Manoj Pandey

**Affiliations:** 1 Department of Surgical Oncology, Institute of Medical Sciences, Banaras Hindu University, Varanasi, 221005 India; 2 SRL Diagnostics, Lanka, Varanasi, India

**Keywords:** Lymphoma, B cell, Large cell, Tuberculosis, Buccal mucosa, Alveolus

## Abstract

**Background:**

Transformation and progression of lymphoma after treatment is well known; however, since the advent of chemotherapy and radiotherapy, progression in untreated lymphoma is seldom seen. We present a case which was misdiagnosed and treated as abdominal tuberculosis later presented with progression and involvement of oral cavity.

**Case presentation:**

A 41-year-old male who presented with urinary symptoms and abdominal pain was diagnosed as abdominal tuberculosis and treated. Two years later, he presented with B symptoms and oral cavity lesion that was diagnosed as diffuse large B cell lymphoma. The patient was treated with R-CHOP chemotherapy with complete regression of the lesion.

**Conclusion:**

Involvement of extranodal sites in predominantly nodal disease does occur; however, involvement of oral cavity is rare. Though primary extranodal lymphoma is reported to occur in oral cavity and oropharynx, natural progression in untreated disease is seldom documented.

## Introduction

Involvement of oral cavity in lymphoma is rare, having a reported incidence of extranodal involvement in 2–5% cases [1]. It is non-Hodgkin’s lymphoma (NHL) which is having more extra nodal spread than Hodgkin’s lymphoma. Extra nodal NHL sites can be GIT, CNS, skin, bone etc., out of which oral cavity as a primary site of extra nodal lymphoma is even rarer [2, 3]. Most of the ulceroproliferative growths in oral cavity in India are squamous cell cancers. There is lack of data reported on the natural course of disease in lymphomas, with only a few reported cases as a primary extra nodal lymphoma in oral cavity sites—tongue, buccal mucosa, and masticator muscles [4]. With this case report, the natural course of diffuse large B cell lymphoma (DLBCL) with the clinical, radiological, and histopathological findings are being discussed as a manifestation in the oral cavity.

## Case report

A 41-year-old male presented to the urology OPD in September 2014 with complaints of colicky pain in the right flank and dysuria of 3 months duration. Examination revealed right renal angle tenderness. The renal function was normal, and an ultrasonography revealed 2.3 × 1.5 cm renal abscess, 1.5 cm left calyceal cyst, 2.8 mm right renal calculus, with a normal bladder, and post-void residual urine (PVRU) of 16 ml. A mild splenomegaly (13 cm), with multiple enlarged lymph nodes in periumbilical region, largest measuring 20 × 19 mm, was also identified; there were no other nodes and no free fluid in the abdomen. A FNAC from lymph nodes was performed which was inconclusive; however, a clinical diagnosis of tuberculosis was made and the patient was started on antitubercular treatment.

**Fig. 1:**
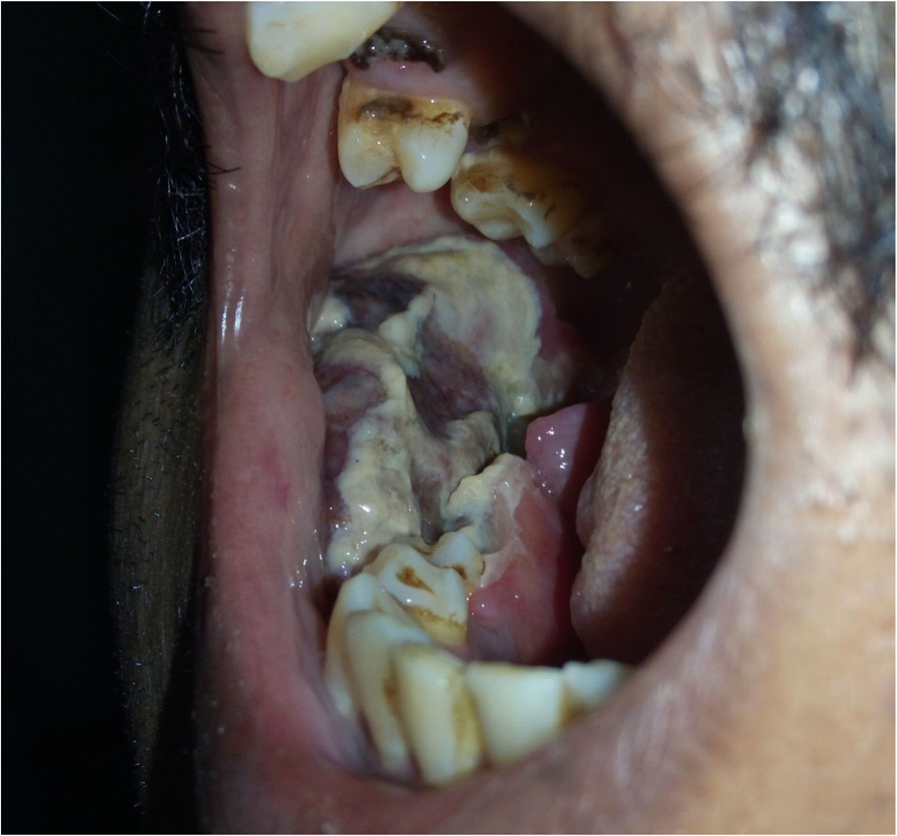
Clinical photograph showing the slough covered, flashy, ulceroproliferative lesion in the buccal mucosa with involvement of alveolus and extension on to right floor of mouth

The patient completed the prescribed antitubercular treatment and remained asymptomatic for 2 years. No follow-up scans were done. In October 2016, the patient again presented to the urology OPD with complaint of recurrent pain in the right flank for 10 days. The pain was moderate in intensity, radiating to the back along with high-grade fever with rigors. There were no urinary complaints this time. Abdominal examination was normal, the renal function was normal, no scans were done, and the patient was referred to orthopedics for treatment of back pain, wherein symptomatic treatment was given. In November 2016, the patient presented to the surgical oncology OPD with complaints of painless progressive ulceroproliferative lesion in the oral cavity, fever, and abdominal pain. On examination of the oral cavity, there was a grayish-brown ulcerative growth starting from the first premolar and continuing till retro molar trigone (RMT) region, involving the right lower gingivobuccal sulcus (GBS), alveolus, and floor of mouth (Fig. 1). Level Ib lymph nodes were palpable in the right neck. The abdominal examination revealed a hard mass in the umbilical and another in the left lumber area with well-defined borders and limited mobility in the lumber mass while the paraumbilical mass was mobile. There were no other significant findings on abdominal and systemic examination.

**Fig. 2:**
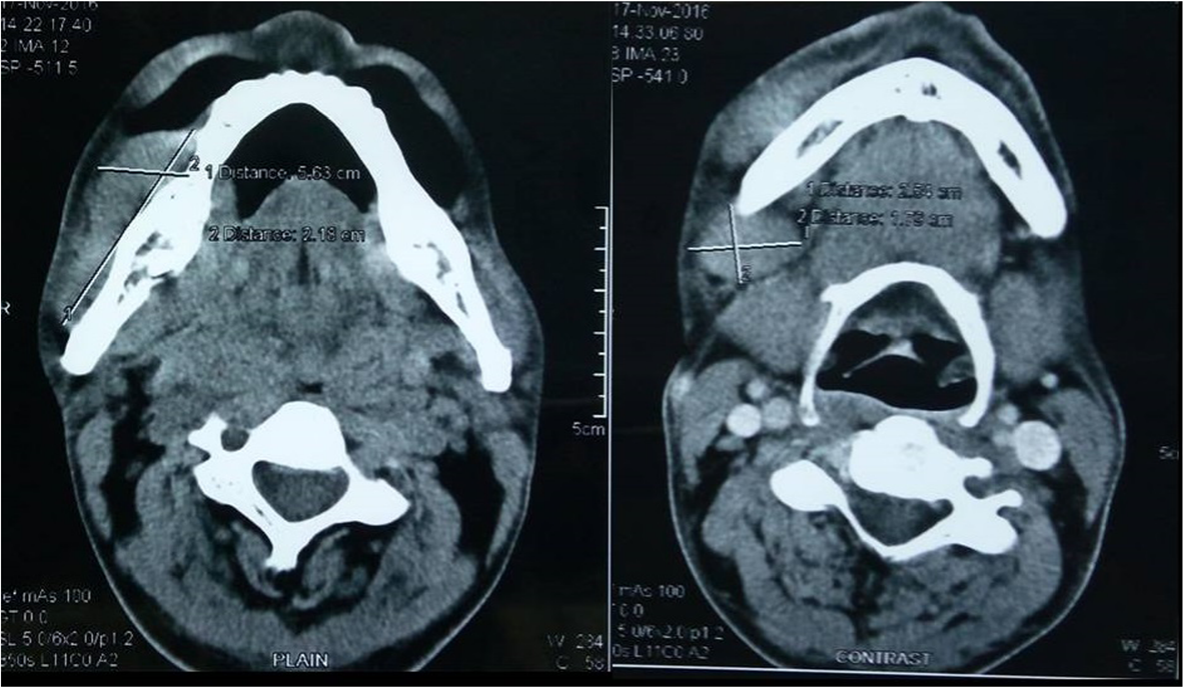
CT scan of the face and neck (Left) showing the lesion in the right buccal mucosa with extension onto the floor of the mouth and destruction of mandible (Right) showing the node in level Ib neck

**Fig. 3:**
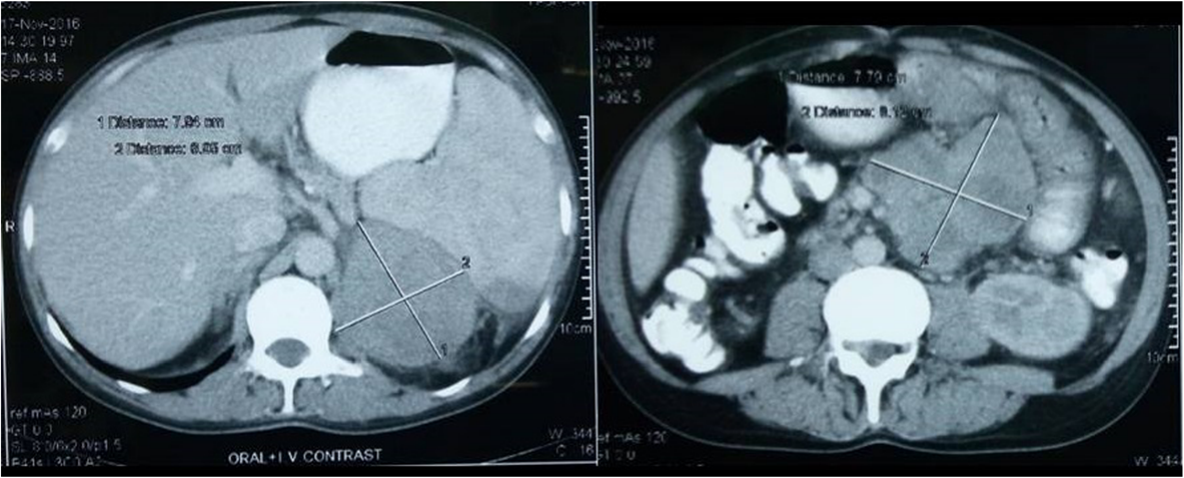
Contrast-enhanced CT scan of the abdomen (Left) showing lesion in the left upper abdomen posterior to the spleen and superior to the left kidney (right) showing matted mass in small bowel mesentery

The CECT of the oral cavity and neck was performed that showed ill-defined thickening in the right inferior gingiva buccal sulcus (premolar and molar region), buccal mucosa, and RMT measuring approximately 54 × 30 × 19 mm; the mass was seen infiltrating the masseter muscle, and destruction of the right inferior alveolus in the molar region and floor of mouth medially was also noted (Fig. 2). Multiple subcentimeter neck nodes level I and II were seen in the CT scan with the largest of size 25 × 18 mm in the right submandibular region. Abdominal CECT showed a matted lymph node mass in the mesentery of the small bowel and another well-defined mass lesion just posterior to the spleen and superior to the left kidney. The left adrenal was not identified separately (Fig. 3).

**Fig. 4:**
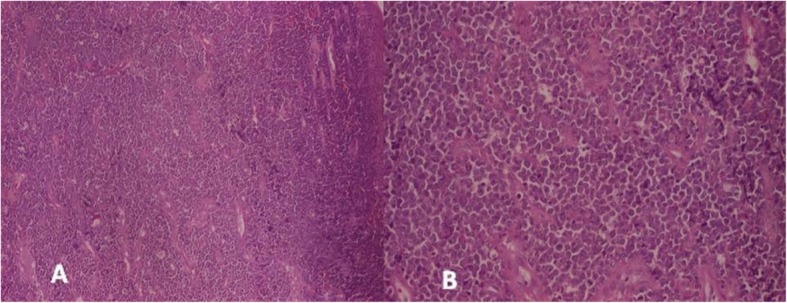
Photomicrograph showing round cell neoplasm. **a** H&E × 40 and **b** H&E × 100

**Fig. 5:**
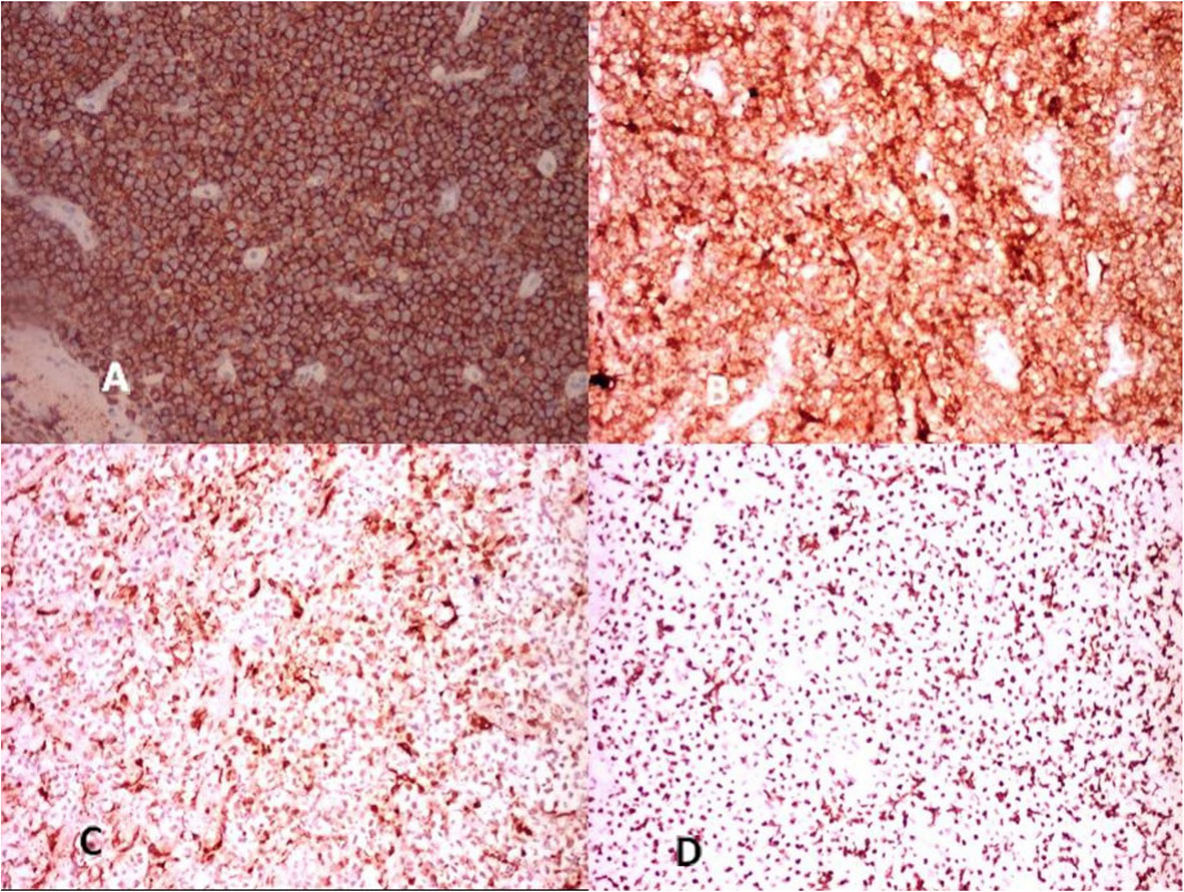
Photomicrograph showing positive staining for **a** CD20 × 40, **b** CD 10 × 40, **c** BCL2 × 40, and **d** Ki-67 × 40

**Fig. 6:**
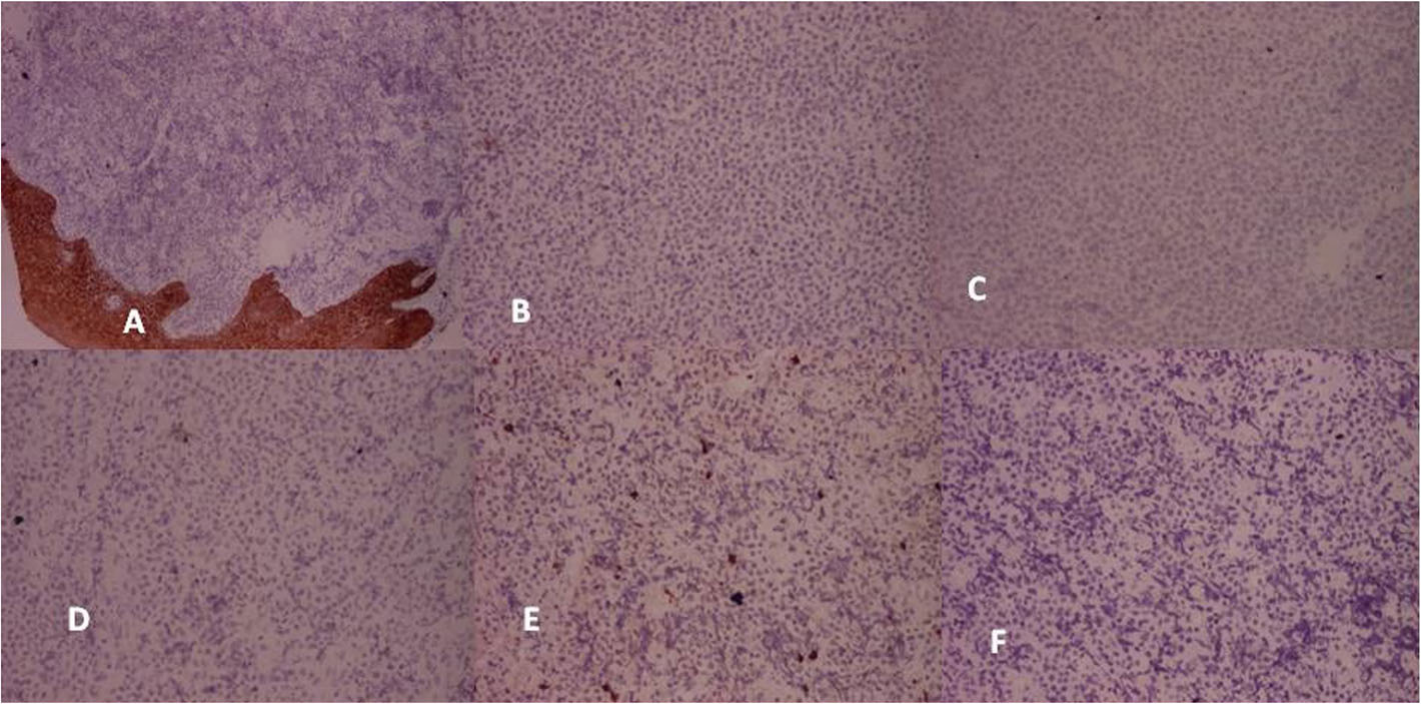
Photomicrograph showing negative staining for **a** cytokeratin × 40, **b** desmin × 40, **c** HMB 45 × 40, **d** ALK1 × 40, **e** CD3 × 40, and **f** CD 99 × 40

A punch biopsy was taken from the right buccal mucosal lesion that showed features suggestive of round cell tumor (Fig. 4). The IHC was performed that showed positivity for CD45, CD 20, CD 10 (immunoreactive score of 4+), and bcl-2 (immunoreactive score of 2+) (Fig. 4). The KI-67 showed immunoreactive score 4+ in > 90% cells (Fig. 5). The CD3, CD 138, CD 99, ALK-1, CK, chromogranin A, desmin, and HMB-45 were negative (Fig. 6). The morphological and IHC features a diagnosis of diffuse large B cell lymphoma (DLBCL). The patient was started on R-CHOP regime and had complete response with regression of the disease after 6 cycles. He continued on maintenance therapy for 1 year and is alive and disease free 14 months after completion of the treatment and stoppage of maintenance therapy.

## Discussion

Non-Hodgkin’s lymphoma of B cell lineage is the third most common malignant lesion in the oral cavity and maxillofacial region. The oral cavity, including the palate, gingiva, tongue, buccal mucosa, floor of mouth, and lips, is the primary site of approximately 2% of extranodal NHL. Waldeyer’s ring is the most frequently involved site, being second only to the GIT in extranodal NHL. Involvement of the muscles and bone is rare in primary extranodal lymphomas [5–7]. Non-immunosuppressed patients of any age can be affected, most patients being in middle age group or older adult, with male preponderance. The most common clinical appearance of NHL in the mouth is a non-healing, painless ulcer [8–10].

Patients with disseminated disease course and the presence of an immunosuppressed condition like AIDS have poor prognosis. EBV and HHV-8 have also been reported to accelerate the process in immunosuppressed patients causing PBL which is a subtype of DLBCL [11].

Since the differentials of an oral lesion are manifold, it becomes mandatory to device good diagnostic methods and representations. Though the morphology of the lesions is different from squamous carcinoma, the spindle cell variant of squamous carcinoma, soft tissue tumors, and melanoma have to be considered as differential diagnosis. Imaging is important for staging the disease, even though it may not have any diagnostic importance as there are no local specific features to suggest a diagnosis of lymphoma. IHC remains the cornerstone of confirmation of diagnosis in such patients. Lymphomas respond very well to chemotherapy, with lesions starting to shrink with the first cycle itself. Hence, inadvertent dental procedures and mutilating surgeries should be avoided especially in patients with disease elsewhere like in the present case and also in cases where there are isolated oral lesions.

There have been a number of case control studies that have found association of chronic infections with lymphoma [12, 13]. The full discussion on the subject is beyond the scope of this study; however, the standard incidence ratio (SIR) has been reported to be ranging from 1.3 to 2.4. The SIR increases with the increase in time since exposure.

As most of the lymphomas are diagnosed and treated at an early stage, it is rare to find cases that demonstrate the natural history of untreated lymphoma.

## Conclusions

Extranodal involvement of the buccal mucosa as part of the natural progression in untreated predominantly nodal lymphoma is never reported before. In patients with oral lesions that have round cell histology, lymphoma should be considered as differential diagnosis. A careful history, radiology, and immunohistochemistry clinches the diagnosis.

## Learning points

Fine needle aspiration cytology is not a reliable investigation in intraabdominal lymphadenopathy.

An inconclusive FNAC should not be taken as negative for malignancy and repeat cytology, and biopsy should be considered.

Tuberculosis should not be the only diagnosis in intraabdominal lymphadenopathy, and other causes of lymphadenopathy should always be considered.

Antitubercular treatment should not be started till the diagnosis of tuberculosis is confirmed with demonstration of AFB or by TB PCR.

Lymphoma responds very well to chemotherapy, and surgery should not be considered as treatment till the lymphoma has been ruled out.

## Data Availability

All the data has been used in this manuscript, and there is no additional data.
